# Inhibition of Wnt signaling pathway suppresses radiation-induced dermal fibrosis

**DOI:** 10.1038/s41598-020-70243-3

**Published:** 2020-08-12

**Authors:** Dong Won Lee, Won Jai Lee, Jaeho Cho, Chae-Ok Yun, Hyun Roh, Hsien Pin Chang, Tai Suk Roh, Ju Hee Lee, Dae Hyun Lew

**Affiliations:** 1 Department of Plastic & Reconstructive Surgery and Institute for Human Tissue Restoration, Yonsei University College of Medicine, 50 Yonsei-ro, Seodaemun-gu, Seoul, 03722 Korea; 2 Department of Radiation Oncology, Yonsei University College of Medicine, Seoul, Korea; 3 Department of Bioengineering and Institute of Nano Science and Technology (INST), College of Engineering, Hanyang University, Seoul, Korea; 4 Department of Dermatology and Cutaneous Biology Research Institute, Yonsei University College of Medicine, Seoul, Korea

**Keywords:** Mechanisms of disease, Experimental models of disease

## Abstract

Progressive fibrosis of the dermal tissues is a challenging complication of radiotherapy whose underlying mechanism is not fully understood, and there are few available treatments. The canonical Wnt/β-catenin signaling pathway plays an important role in fibrosis as well as in the epithelial-to-mesenchymal transition (EMT). We investigated whether inhibition of Wnt/β-catenin signaling with sLRP6E1E2, a molecule that binds to extracellular Wnt ligands, ameliorated radiation-induced fibrosis both in vitro and in vivo. Radiation with a single dose of 2 Gy not only facilitated fibrosis in cultured human dermal fibroblasts via activation of the Wnt/β-catenin pathway but also initiated EMT in cultured keratinocytes, developing collagen-producing mesenchymal cells. sLRP6E1E2-expressing adenovirus treatment exerted anti-fibrotic activity in irradiated cultured dermal fibroblasts and keratinocytes. In a mouse model, a single fraction of 15 Gy was delivered to the dorsal skins of 36 mice randomized into three groups: those receiving PBS, those receiving control adenovirus, and those receiving decoy Wnt receptor-expressing adenovirus (dE1-k35/sLRP6E1E2). The mice were observed for 16 weeks, and excessive deposition of type I collagen was suppressed by sLRP6E1E2-expressing adenovirus treatment. These results demonstrate that the modulation of the Wnt/β-catenin pathway has the potential to decrease the severity of radiation-induced dermal fibrosis.

## Introduction

Radiotherapy is an effective treatment for controlling solid tumors such as head and neck cancer, breast cancer, and soft tissue sarcoma. However, collateral injury to the surrounding healthy tissues may result in toxicity and frequently limits the therapeutic dose of radiation that can be delivered^1^. Since the skin is usually the first site of entry of radiation, various skin reactions can occur. In the acute phase of radiation injury, erythema may be evident within hours of irradiation, and desquamation and ulceration appear at higher doses. These acute events can be addressed symptomatically and generally resolve without exacerbation^2^. However, progressive fibrosis of the dermal tissues is a late adverse effect of radiation that is not always resolved. Pain, limited range of motion, and poor cosmesis are well-described adverse events attributed to radiation-induced fibrosis of the skin^3^. Unfortunately, there are few anti-fibrotic therapies available.

Tissue fibrosis is the excessive accumulation of collagen and other extracellular matrix components following a breakdown in the normal balance of extracellular matrix synthesis and degradation^4^. Several mediators are involved and act on fibroblasts to cause tissue fibrosis. In addition to the TGF-β signaling pathway, which is a key mediator of fibroblast activation, the Wnt/β-catenin signaling pathway plays an important role in fibrotic reactions^5^. In the setting of radiation, β-catenin mediates the effects of radiation in fibroblasts, and its modulation has the potential to decrease the severity of radiation-induced complications^4^.

In this study, we tested the hypothesis that inhibition of the Wnt/β-catenin signaling pathway would ameliorate radiation-induced fibrosis. Inhibition of the pathway was accomplished using adenovirus gene therapy expressing a Wnt antagonist that binds Wnt ligands to block interactions with receptors^6^. Low-density lipoprotein receptor-related protein 6 (LRP6), a Wnt ligand, is required for activation of the canonical Wnt/β-catenin pathway^7^. A novel soluble Wnt receptor, sLRP6E1E2, which is composed of the LRP6 E1 and E2 regions, was used to inhibit the pathway^8^. Therefore, we explored the biological effects of the binding of sLRP6E1E2 to extracellular Wnt ligands after irradiation.

## Results

### Decoy Wnt receptor sLRP6E1E2 inhibits Wnt/β-catenin pathway in irradiated cells

**Figure 1:**
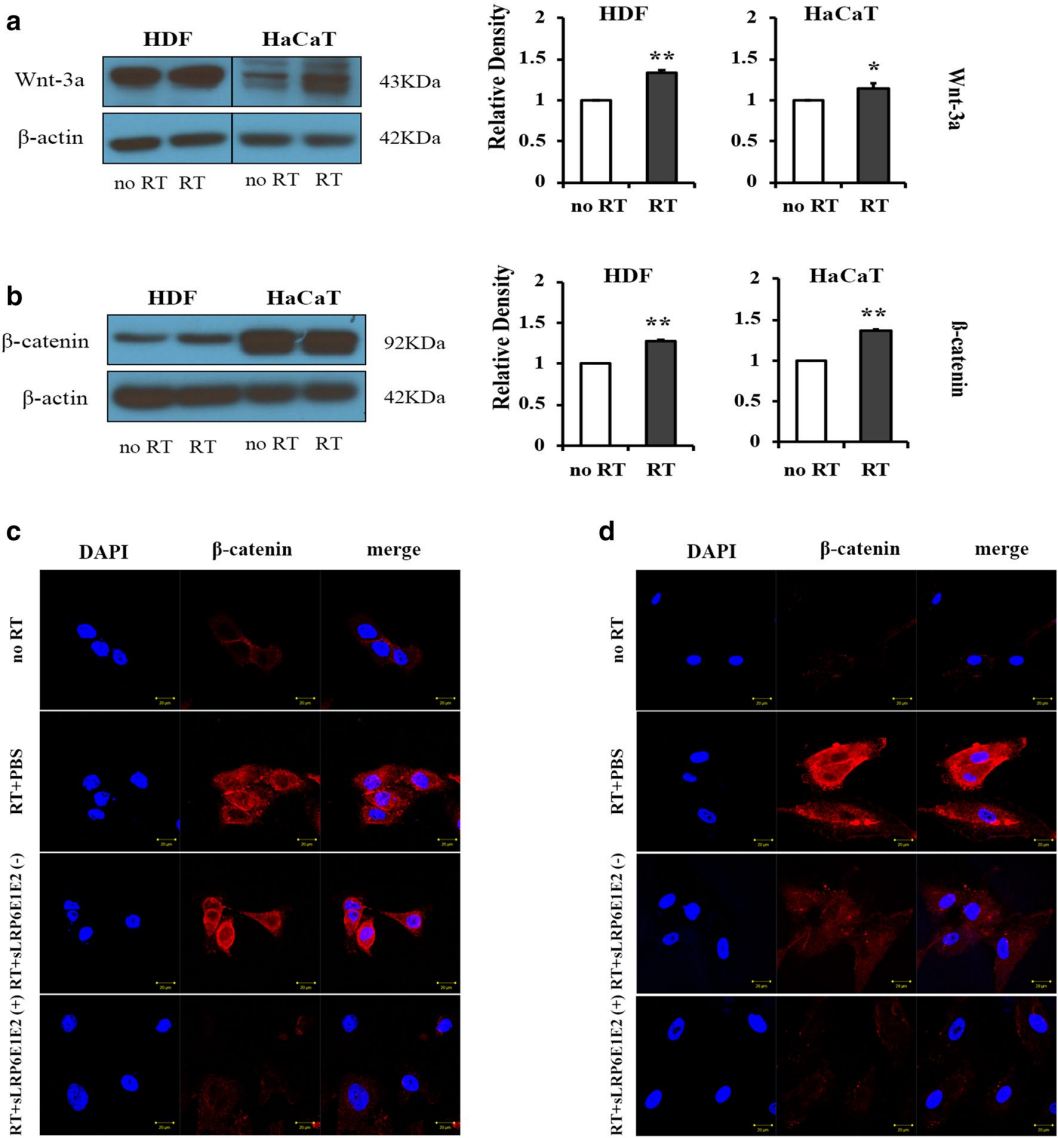
Increased Wnt-3a and β-catenin in irradiated cells and inhibition of Wnt/β-catenin pathway by decoy Wnt receptor sLRP6E1E2. Human dermal fibroblasts (HDFs) and HaCaT keratinocytes were irradiated, and cell lysates were immunoprecipitated with antisera against (**a**) Wnt-3a and (**b**) β-catenin. No IR: not irradiated; IR: irradiated; *p < 0.05; **p < 0.01. (**c**) HDF and (**d**) HaCaT keratinocytes were transduced with dE1-k35/sLRP6E1E2 or dE1-k35 after radiation. Cells were labeled with anti- β-catenin to test intranuclear translocation.

Dermal fibroblasts and keratinocytes, representative cells that compose the skin, were selected to determine the effect of irradiation on skin tissues. We began with the premise that the Wnt/β-catenin signaling pathway would be activated in the setting of radiation. To verify that radiation contributed to the activation of the Wnt/β-catenin pathway, we examined Wnt-3a and β-catenin expression level in human dermal fibroblasts (HDF) and HaCaT keratinocytes at 48 h after a single dose of 2-Gy irradiation. Both Wnt3a and β-catenin are upregulated at the protein level (Fig. 1a,b). This suggests that radiation is responsible for high expression of Wnt3a and its effector β-catenin protein in irradiated fibroblasts and keratinocytes.

It has been previously reported that activation of Wnt/β-catenin pathway elicits reduced β-catenin localization at the plasma membrane and increased β-catenin levels in the cytosol and nucleus^6^. Therefore, we examined the extent of cytosolic and nuclear localization of β-catenin by immunofluorescence staining of irradiated HDFs and HaCaT keratinocytes after transduction with dE1-k35/sLRP6E1E2 (sLRP6E1E2-expressing virus) or dE1-k35 (control virus). In absence of radiation, β-catenin was rarely observed in the cytosol and nucleus. Upon radiation, control groups (radiation + PBS and radiation + sLRP6E1E2 (−) virus) showed increased β-catenin localization on the cytosol and nucleus, while dE1-k35/sLRP6E1E2-transduced cells showed lower levels of nuclear localization of β-catenin than control groups (Fig. 1c,d). These data suggest that decoy Wnt receptor sLRP6E1E2 inhibits Wnt/β-catenin pathway in irradiated fibroblasts and keratinocytes. It was also supported by the results of previous studies using the same viruses^6,9^.

### Decoy Wnt receptor sLRP6E1E2 inhibits TGF-β/Smad pathway and suppresses collagen production in irradiated HDFs

**Figure 2:**
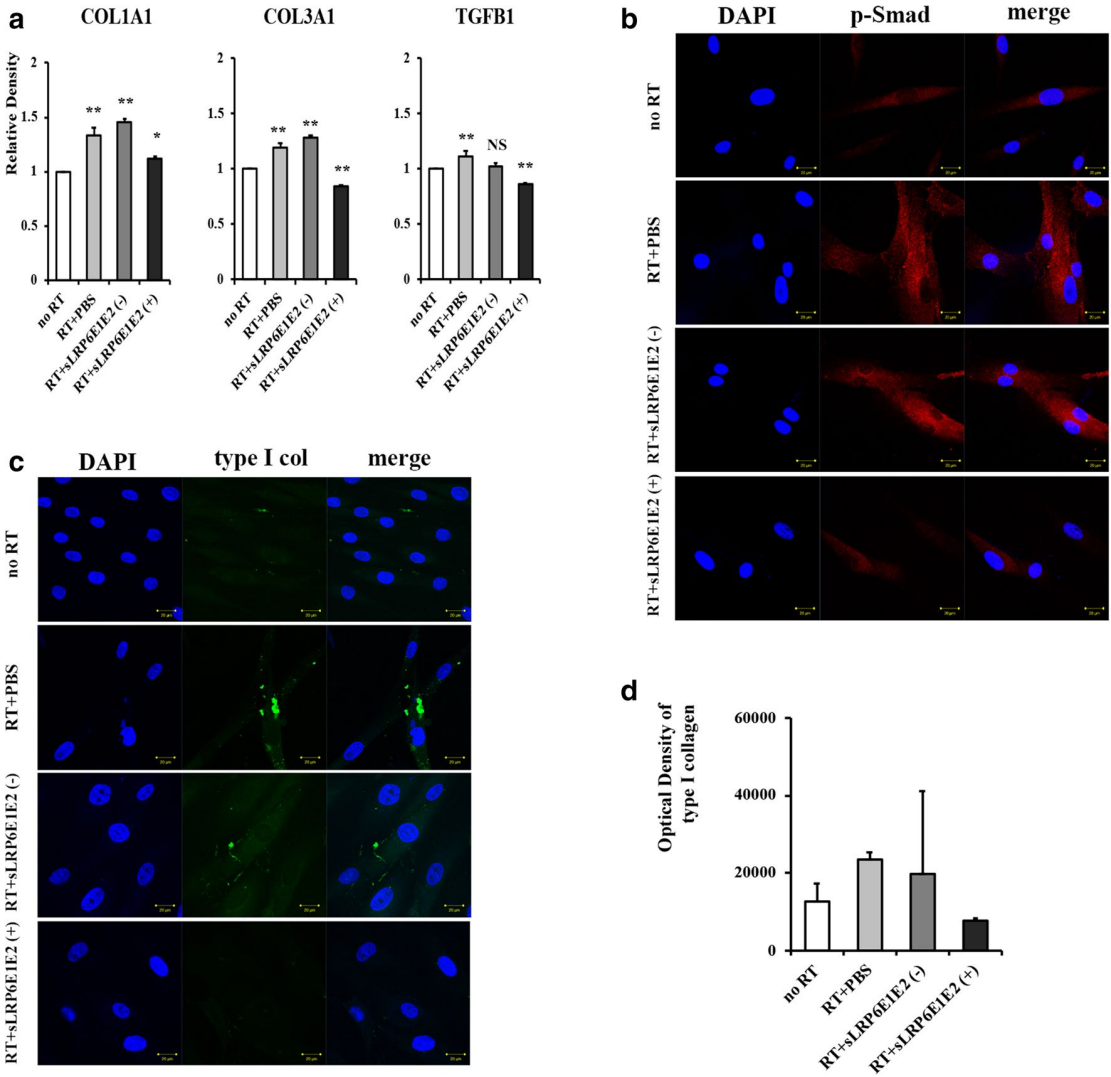
Inhibition of Wnt/β-catenin pathway by decoy Wnt receptor sLRP6E1E2 in irradiated human dermal fibroblasts (HDFs), and decreased production of collagen type I. (**a**) Quantitative mRNA levels of type-I, -III collagen and TGF-β in HDFs were assessed by qRT-PCR after transduction with dE1-k35/sLRP6E1E2 or dE1-k35. *p < 0.05; **p < 0.01 vs no radiation. Irradiated HDFs were transduced with dE1-k35/sLRP6E1E2 or dE1-k35, and immunocytochemical staining for (**b**) phospho-Smad 2/3 (red) and (**c**) type I collagen (green) was performed. DAPI: blue. (**d**) The expression levels of type I collagen were assessed semi-quantitatively using MetaMorph imaging analysis software.

The Wnt/β-catenin pathway regulates a wide range of cellular functions including fibrosis^6,10^. To test the potential effect of sLRP6E1E2 on in vitro fibrosis by inhibition of Wnt3a/β-catenin signaling in irradiated HDFs, mRNA expression of collagen type I α1, collagen type III α1 and TGF-β1 was examined, and immunocytochemistry for phospho-Smad 2/3 and type I collagen was performed in irradiated HDFs after treatment with dE1-k35/sLRP6E1E2 or dEl-k35. A single dose of 2-Gy irradiation was given to HDF, and the results were collected after 48 h. The mRNA expression of the genes encoding type I α1 and III α1 collagen was reduced by 23% and 34%, respectively, in HDFs transfected with dE1-k35/sLRP6E1E2 compared to levels in dE1-k35-transfected controls (Fig. 2a). In particular, type III α1 collagen expression was dramatically reduced compared to the level in non-irradiated cells. The mRNA expression of the gene for TGF-β1 in HDF was increased in control groups (radiation only and sLRP6E1E2 (−) control virus), while it was reduced in sLRP6E1E2-expressing virus (Fig. 2a,b). According to immunocytochemical staining for type I collagen in HDFs treated with sLRP6E1E2, type I collagen fibers were not observed (Fig. 2c). Taken together, these findings suggest that sLRP6E1E2-expressing viruses suppress radiation-induced fibrosis in HDFs.

### Decoy Wnt receptor sLRP6E1E2 inhibits epithelial-to-mesenchymal transition (EMT) and suppresses collagen production in irradiated HaCaT keratinocytes

**Figure 3:**
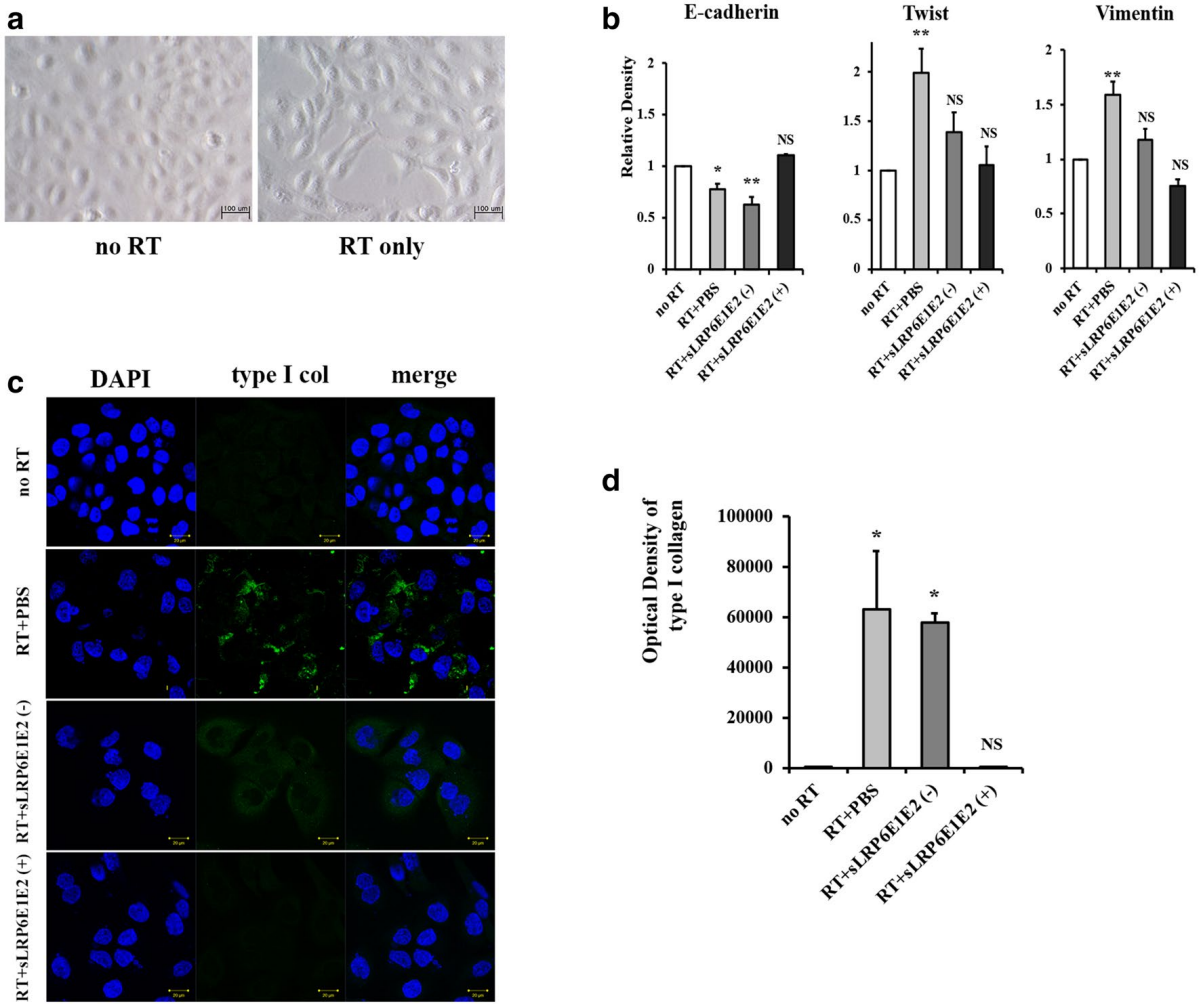
Inhibition of epithelial-to-mesenchymal transition by decoy Wnt receptor sLRP6E1E2 in irradiated HaCaT keratinocytes, and decreased production of collagen type I. (**a**) Under phase-contrast microscopy, spindle-shaped HaCaT keratinocytes were observed after irradiation. (**b**) The mRNA expression of the gene encoding E-cadherin (epithelial marker) increased, while levels of those encoding twist and vimentin decreased (mesenchymal markers). *p < 0.05; **p < 0.01 vs no radiation. (**c**) After transduction with dE1-k35/sLRP6E1E2 or dE1-k35, Cells were stained with DAPI (blue) and anti-type I collagen (green). (**d**) The expression levels of type I collagen were assessed semi-quantitatively using MetaMorph imaging analysis software. *p < 0.05 vs no radiation.

EMT is known to be an important process in fibrosis^4^, and the Wnt/β-catenin pathway also plays an important role in this process^6^. We hypothesized that irradiated keratinocytes would be converted into collagen-producing mesenchymal cells by the mechanism of EMT, and converted mesenchymal cells ultimately produce collagen fibers. In irradiated HaCaT keratinocytes, morphological changes were observed, as they adopted a spindle-shaped appearance like fibroblasts (Fig. 3a). Figure 3b shows that irradiated HaCaT keratinocytes exhibited downregulation of mRNA encoding the epithelial marker E-cadherin. Conversely, mesenchymal markers (i.e., twist and vimentin) were markedly upregulated in irradiated keratinocytes, suggesting that EMT occurred. Furthermore, the results of immunocytochemistry for type I collagen demonstrated that irradiated HaCaT keratinocytes eventually produced type I collagen fibers (Fig. 3c). These results suggest that irradiated keratinocytes changed into collagen-producing fibroblasts functionally and morphologically.

We investigated whether sLRP6E1E2-expressing viruses inhibit the EMT process and collagen synthesis in irradiated keratinocytes. We found that the mRNA levels of most markers, whether epithelial or mesenchymal, were not significantly altered in sLRP6E1E2-treated cells from those in non-irradiated cells, contrary to the results of the control groups (radiation + PBS and radiation + sLRP6E1E2 (−) virus) (Fig. 3b), indicating that EMT in irradiated keratinocytes was inhibited by treatment with dE1-k35/sLRP6E1E2 viruses. Based on immunocytochemical staining for type I collagen, collagen fibers were not produced in irradiated keratinocytes treated with sLRP6E1E2, while stained type I collagen was observed in control groups (Fig. 3c).

### Decoy Wnt receptor sLRP6E1E2 inhibits Wnt/β-catenin pathway in irradiated mouse

**Figure 4:**
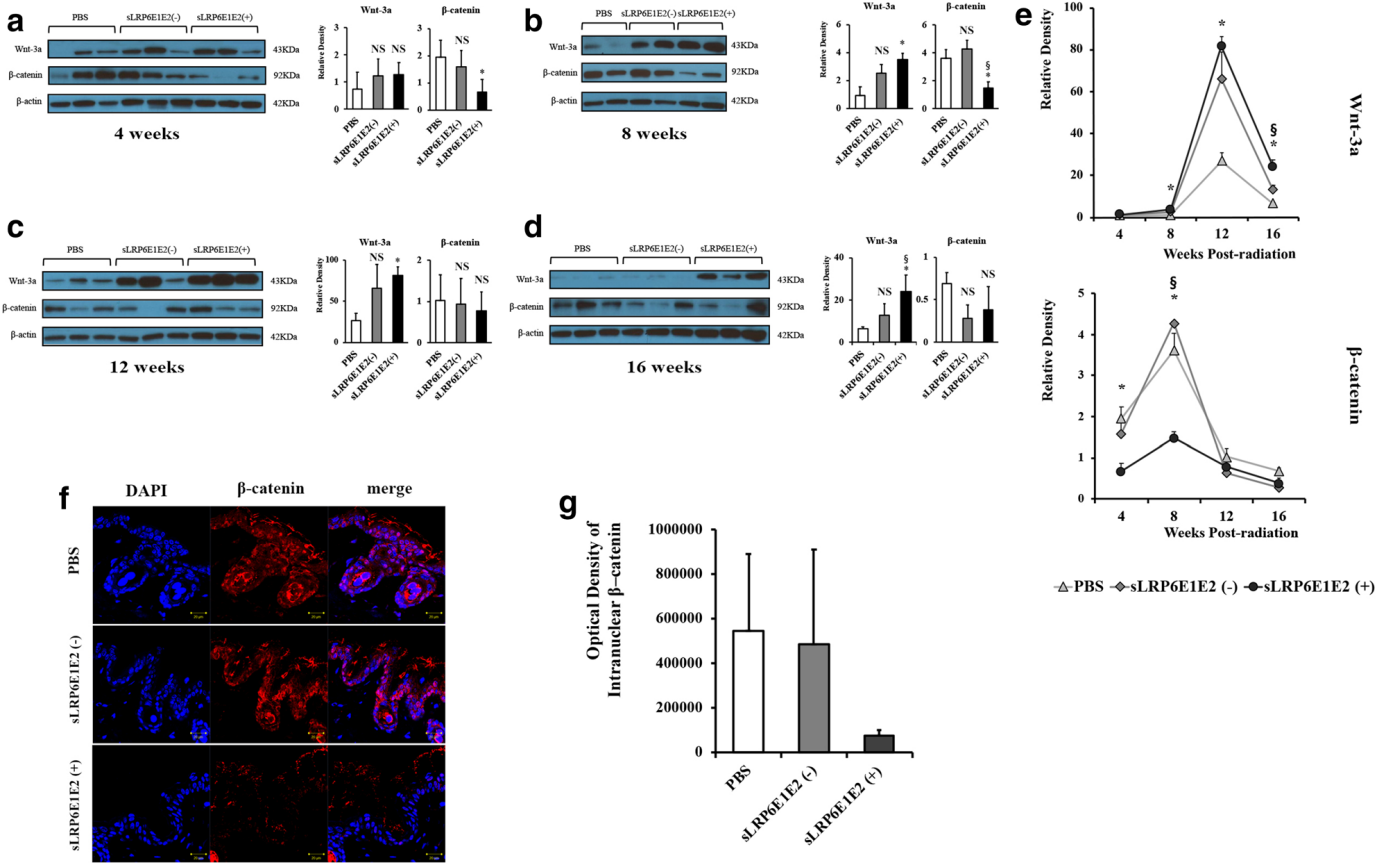
Inhibition of Wnt/β-catenin pathway by decoy Wnt receptor sLRP6E1E2 in irradiated mouse. Wnt-3a and β-catenin were detected by western blot after transduction with dE1-k35/sLRP6E1E2 or dE1-k35. The blots were from one gel and were then probed with different antibodies one at a time. Each blot was taken from one mouse. β-actin was used as an internal standard. *p < 0.05 vs PBS. ^§^p < 0.05 vs sLRP6E1E2 (–). (**a**–**d**) Gels/ blots and quantification at post-radiation 4, 8, 12, and 16 weeks, respectively. (**e**) Relative density of Wnt-3a and β-catenin compared to that of control protein as detected using densitometry at various post-radiation time points (**a**–**d**). (**f**) Cells were labeled with anti- β-catenin to test intranuclear translocation at 8 weeks post-radiation. (**g**) The expression levels of intranuclear β-catenin were assessed semi-quantitatively using MetaMorph imaging analysis software.

We next evaluated changes in the Wnt/β-catenin pathway in the setting of radiation in a mouse model treated with PBS, control adenovirus (dE1-k35), or decoy Wnt receptor-expressing adenovirus (dE1-k35/sLRP6E1E2) using western blot analysis of Wnt-3a and β-catenin. In the PBS control, levels of Wnt-3a began increasing after irradiation and peaked at 12 weeks, while β-catenin increased rapidly at 4 weeks, peaked at 8 weeks, and decreased 12–16 weeks after irradiation (Fig. 4a–e). This suggests that radiation activates the Wnt/β-catenin pathway during the initial 12 weeks, subsiding after 16 weeks post-radiation. When treated with sLRP6E1E2-expressing virus, the rate of increase and the peak in the protein expression of Wnt-3a were markedly pronounced. However, expression of the β-catenin protein was reduced, with a lower peak than that in mice treated with PBS control or dE1-k35 control (Fig. 4a–e). These findings suggest that the level of β-catenin in the dE1-k35/sLRP6E1E2 group generally decreases owing to the action of sLRP6E1E2, which suppresses the Wnt/β-catenin pathway, while the level of Wnt-3a in the same group is elevated to compensate for the suppression of the Wnt/β-catenin pathway via negative feedback.

Figure 4f shows whether β-catenin translocates to the nucleus in each group at 8 weeks post-radiation. Control groups (PBS and sLRP6E1E2 (−)) showed that β-catenin was mainly located in the nucleus, whereas reduced levels of β-catenin in the cytosol and nucleus were noted in sLRP6E1E2-expressing adenovirus treatment group. This suggests that Wnt/β-catenin pathway was suppressed in dE1-k35/sLRP6E1E2-transfected mouse.

### Decoy Wnt receptor sLRP6E1E2 suppresses collagen production in irradiated mouse

Through a preliminary study of the dose-dependent response to radiation in a mouse model, the appropriate dose of radiation for the current study was determined to be a single fraction of 15 Gy, as a higher dose of radiation induced ulceration of the skin that activated wound healing and fibrosis processes (data not shown). As we sought to focus on radiation-induced fibrosis in this study, fibrosis caused by wound healing should be excluded. After 15 Gy radiation was delivered to the dorsal skin, tiny dry desquamations appeared in approximately 1/3 of the mice in each group within 4 weeks, and fields receiving radiation healed without ulceration in all mouse. Upon examination by planimetric analysis and laser Doppler flowmetry, there were no significant differences between groups in almost values (Supplementary Fig. S1). This indicates that the radiation dose of 15 Gy may not cause tissue contracture within the experimental period, although radiation-induced fibrosis may occur. A single fraction of 15 Gy is thought to be similar to that used in clinical settings in humans.

**Figure 5:**
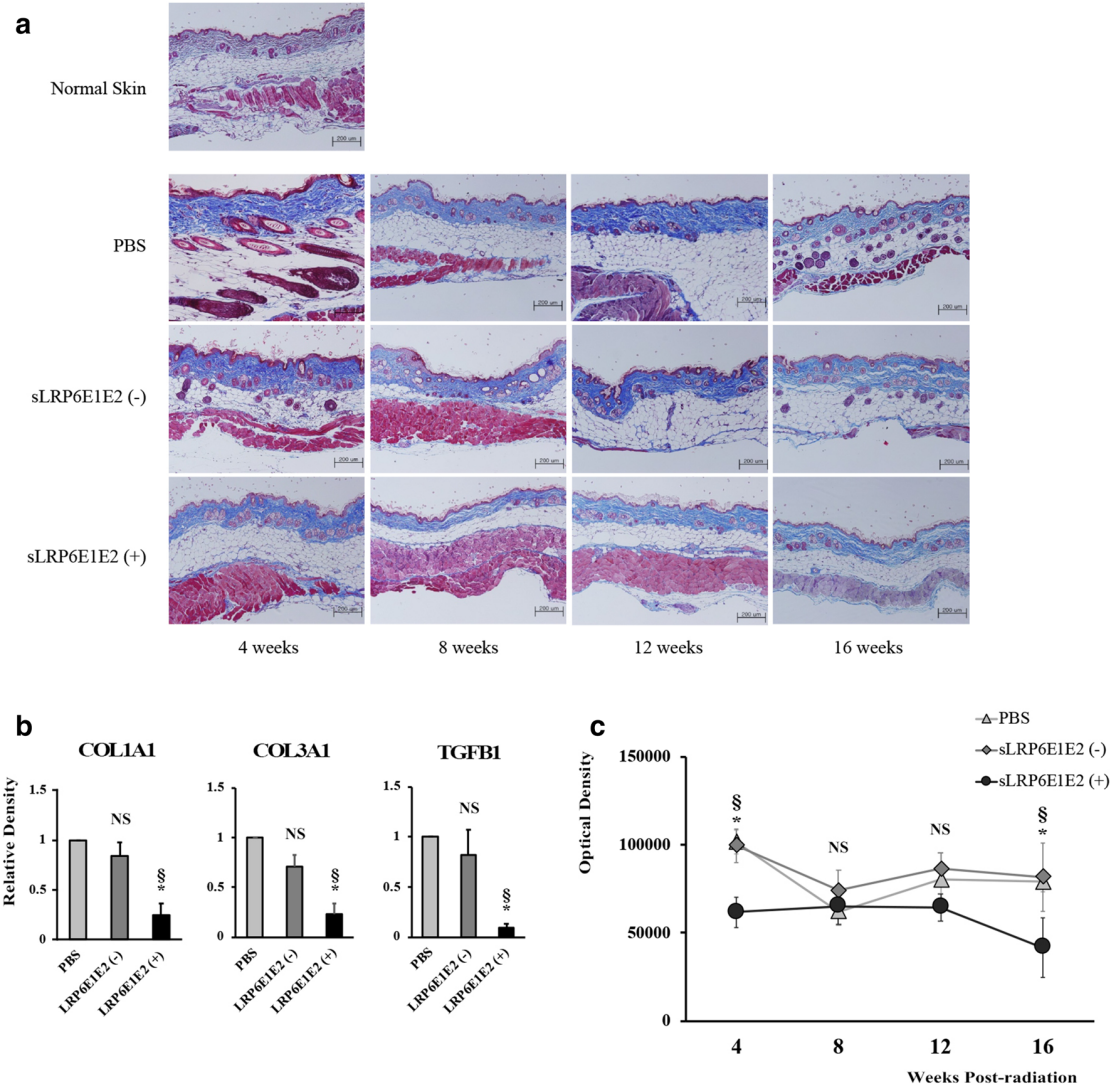
Anti-fibrotic effect of decoy Wnt receptor sLRP6E1E2 in irradiated mouse. (**a**) Histological views of non-irradiated normal tissue and irradiated tissues of PBS control group, control virus (dE1-k35) group, and sLRP6E1E2-treated group by Masson’s trichrome staining. The mice in the sLRP6E1E2-treated group showed a thinner dermis and many miniaturized follicles, while mice in control groups have thicker dermis tissue and adnexa replaced by collagenous fibers. (**b**) Quantitative mRNA levels of type-I, -III collagen and TGF-β in HDFs were assessed. (**c**) Semi-quantitative analysis of type I collagen immunohistochemistry. The amount of collagen in the sLRP6E1E2-treated group was not significantly different from that of non-radiated tissues over the entire follow-up period. *p < 0.05 vs PBS. ^§^p < 0.05 vs sLRP6E1E2 (−).

According to Masson’s trichrome staining, layers of dermal collagen fibers generally increased and thickened in irradiated groups (PBS, sLRP6E1E2 (−), and sLRP6E1E2 (+) groups) compared to those in normal dermal tissue. Thus, radiation seems to be responsible for increasing collagenous fibrosis. Among the irradiated groups, a relatively thin dermis and many miniaturized follicles were observed in the sLRP6E1E2-treated group, while mice in the control groups (PBS, sLRP6E1E2 (−)) had thicker dermal tissues and fewer follicles. In addition, adnexa were replaced by collagenous fibers in both control groups (Fig. 5a).

To test the potential effect of sLRP6E1E2 on in vivo fibrosis, mRNA expression of collagen type I α1, collagen type III α1 and TGF-β1 was examined at 12 weeks post-radiation, and immunohistochemistry for type I collagen was performed at each time point. The mRNA expression of collagen type I α1, collagen type III α1 and TGF-β1 was significantly reduced in dE1-k35/sLRP6E1E2-transfected mouse compared to controls (Fig. 5b). The results of collagen type I immunohistochemistry were analyzed using MetaMorph image for semi-quantitative analysis. In the sLRP6E1E2-treated group, there was a decrease in collagen expression during the follow-up period. Especially, the amount of type I collage was significantly different between sLRP6E1E2-treated group and both control groups at 4 and 16 weeks post-radiation (Fig. 5c). The lack of an increase in the expression of type 1 collagen in the sLRP6E1E2-treated group suggests that the Wnt/β-catenin pathway plays an important role in radiation-induced fibrosis.

## Discussion

We investigated whether inhibition of the canonical Wnt/β-catenin pathway could reverse radiation-induced fibrosis. sLRP6E1E2-expressing adenovirus was used to inhibit a Wnt ligand-dependent process. In an irradiated mouse model, an elevation in the β-catenin level was verified, and we found that overproduction of type I collagen, as a representative of extracellular matrix components, could be controlled by inhibition of the Wnt/β-catenin pathway. This indicates that inhibition of the β-catenin mediator is effective in ameliorating radiation-induced fibrosis.

Several pathways involved in fibrosis from initial stimulus to collagen synthesis offer various therapeutic targets. Among these, we selected the Wnt/β-catenin pathway as a target for controlling radiation-induced fibrosis, although TGF-β is known to be a key factor in the fibrotic reaction^11^. Reactive oxygen species generated by radiation exposure are immediate activators of TGF-β, which then initiates the process of upregulated collagen synthesis^12^. Recent studies suggest that the effect of TGF-β on wound healing is mediated by β-catenin, and a similar process of Wnt/β-catenin signaling might contribute to radiation-induced fibrosis^13–15^. We hypothesized that inhibiting β-catenin would significantly mitigate radiation-induced fibrosis in dermal tissues.

Before developing a strategy to reduce the progression of radiation-induced fibrosis, we should closely consider both the starting and ending points of the treatment. Radiation-induced fibrosis presents in the late phase, sometimes even decades after treatment. The perpetuation of the fibrotic reaction may not require continued paracrine stimulation by fibrogenic cytokines but may rely on positive autocrine signaling^4^. It is postulated that collagen synthesis is subsequently continued by autocrine induction of connective tissue growth factor^16,17^. Whether early activation of TGF-β is relevant to the late effect of radiation-induced fibrosis has not been confirmed. However, several studies have demonstrated the amelioration of radiation-induced fibrosis via the early inhibition of the TGF-β pathway^18,19^. In the present in vivo study, the results of the control groups revealed that the protein levels of Wnt-3a and β-catenin began increasing after irradiation with a peak at 12 and 8 weeks respectively and decreased at 12–16 weeks, suggesting that the activation of the Wnt/β-catenin pathway is confined to within 16 weeks post-radiation. The synthesis of type I collagen in the mouse model increased even in the early phase, although radiation-induced fibrosis is known as a late effect in humans. The current result is consistent with previous reports showing the early deposition of collagen fibers in humans^20,21^. In this respect, radiation-induced fibrosis in the dermis clinically manifests as a late effect even though collagen deposition starts immediately after irradiation. Therefore, we applied the adenovirus treatment until 11 weeks post-irradiation, when β-catenin activation subsides.

In the past, it was believed that locally resident mesenchymal cells were the only source of collagen-producing cells, but other origins of fibroblasts have now been identified. These are epithelial and endothelial cells capable of undergoing EMT and endothelial–mesenchymal transition (EndoMT)^22^. Circulating fibrocytes from bone marrow are another circulating fibroblast precursor^23^. We focused on EMT as a source of fibroblasts. The Wnt/β-catenin pathway plays an important role in the EMT process as well as the fibrotic reaction^6^. It has been proposed that epithelial cells in dermal tissues may transform into fibroblasts, and we assumed that inhibition of the Wnt/β-catenin pathway would decrease such an EMT process. Accordingly, fibrotic activity was expected to be suppressed by decreasing the number of fibroblasts. In vitro analysis confirmed this hypothesis, as irradiated keratinocytes produced collagen fibers and collagen synthesis was blocked by the adenovirus treatment. In summary, inhibition of the Wnt/β-catenin pathway may ameliorate radiation-induced fibrosis via two mechanisms: direct suppression of fibrogenic activity and indirect suppression by reducing the number of fibroblasts through suppression of an EMT process.

In conclusion, radiation facilitates the fibrotic reaction in cultured keratinocytes as well as cultured human dermal fibroblasts, while sLRP6E1E2 exerts anti-fibrotic activity in irradiated cultured cells (in vitro study). In a mouse model, excessive collagen deposition was controlled in the sLRP6E1E2-treated group (in vivo study). Taken together, these results support the idea that modulation of the Wnt/β-catenin pathway has the potential to decrease the severity of radiation-induced dermal fibrosis.

## Methods

### Cell culture and in vitro irradiation

HDF and HaCaT keratinocytes (American Type Culture Collection, Manassas, VA, USA) were seeded at passage 4 in 6-well plates and grown in a complete cell culture medium consisting of Dulbecco’s modified Eagle medium (DMEM) with 100 U/ml penicillin, 100 g/ml streptomycin sulfate, and 10% fetal bovine serum (FBS) at 37 °C in a humidified atmosphere containing 5% CO_2_ until forming a confluent monolayer. An X-RAD320 irradiator (Precision X-Ray, North Branford, CT, USA) was used to irradiate cell cultures with a single dose of 2-Gy radiation.

### Treatment with adenovirus-expressing decoy Wnt receptor

The virus dE1-k35/sLRP6E1E2 was obtained as previously described^6^. The replication-incompetent dE1-k35 was used as a negative control^8^. Titers of generated adenoviruses were determined using the optical density of the viral genome. For both dE1-k35/sLRP6E1E2 (sLRP6E1E2-expressing virus) and dE1-k35 (control virus), a multiplicity of infection (MOI) of 100% was transfected into cultured dermal fibroblasts and HaCaT keratinocytes. Irradiation of cell cultures was followed immediately by viral transfection.

### Quantitative real-time reverse transcriptase-polymerase chain reaction (qRT-PCR)

To evaluate mRNA levels of collagen type 1 α1 (*COL1A1*), collagen type 3 α1 (*COL3A1*), TGF-β1 (*TGFB1*) in cultured HDF cells and those of E-cadherin (*CDH1*), vimentin (*VIM*), and twist (*TWIST1*) in cultured HaCaT keratinocytes, qRT-PCR was performed 48 h after irradiation. Total RNA was prepared with the RNeasy Mini Kit (Qiagen, Hilden, Germany), and complementary DNA was prepared from 0.5 µg of total RNA by random priming using a first-strand cDNA synthesis kit (AccuPower RT PreMix, Bioneer, Daejeon, Korea) under the following conditions: 95 °C for 5 min, 37 °C for 2 h, and 75 °C for 15 min. Applied Biosystems TaqMan primer/probe kits were used to analyze mRNA expression levels with an ABI Prism 7,500 HT Sequence Detection System (Applied Biosystems, Foster City, CA, USA). For cDNA amplification, AmpliTaq Gold DNA polymerase was activated by 10-min incubation at 95 °C; this was followed by 40 cycles of 15 s at 95 °C and 1 min at 60 °C for each cycle. The mRNA expression levels were normalized to the levels of the *GAPDH* housekeeping gene, and then relative quantities were expressed as fold-inductions compared with the control gene after determining the threshold cycle and drawing standard curves. The following primers were used: Hs00164004_m1 (COL1A1), Hs00164103_m1 (COL3A1), Hs00998133_m1 (*TGFB1*), Hs 01023894_m1 (*CDH1*), Hs 00185584_m1 (*VIM*), Hs 00361186_m1 (*TWIST1*), and Hs99999905_m1 (GAPDH, reference).

### Western blot analysis

Western blot analysis was performed to examine proteins associated with the Wnt/β-catenin signaling pathway in cultured cells and animal tissues. Cultured cells or tissues were lysed in 50 mM Tris–HCl (pH 7.6), 1% Nonidet P-40, 150 mM sodium chloride, and 0.1 mM zinc acetate in the presence of protease inhibitors. Protein concentration was determined by the Lowry method (Bio-Rad, Hercules, CA, USA), and 20 µg of each sample was separated by 10% sodium dodecyl sulfate polyacrylamide gel electrophoresis. The gels were then transferred electrophoretically onto a polyvinylidene difluoride membrane (Millipore, Billerica, MA, USA). The membrane was blocked with blocking buffer for 1 h and then incubated overnight at 4 °C with primary antibodies against β-catenin (Cell Signaling Technology, Beverly, MA, USA), Wnt (Santa Cruz Biotechnology, Inc., Santa Cruz, CA, USA), and actin (mouse monoclonal, Sigma-Aldrich, St. Louis, MO, USA). After a 2-h incubation with the secondary antibodies horseradish peroxidase-conjugated rabbit antibody (Santa Cruz Biotechnology) and horseradish peroxidase-conjugated mouse antibody (Santa Cruz Biotechnology), protein bands were visualized using an ECL detection kit (Thermo, Fisher Scientific, Waltham, MA, USA) according to the manufacturer’s instructions. Protein expression was analyzed using ImageJ software (National Institutes of Health, Bethesda, MD, USA).

### Morphology of irradiated HaCaT keratinocytes

After irradiation, HaCaT keratinocytes were cultured for 48 h, and phenotypic changes were examined using phase-contrast microscopy (Olympus, Tokyo, Japan).

### Immunocytochemistry for type I Collagen, phospho-Smad and β-catenin

After 48 h post-radiation, cultured cells were washed twice with phosphate-buffered saline (PBS), fixed in 4% paraformaldehyde for 15 min at room temperature, and then permeabilized by incubation for 15 min with 0.01% Tween 20 in PBS. Samples were blocked with 5% bovine serum albumin followed by incubation with anti-collagen I, anti-phospho-Smad 2/3 and anti-β-catenin (1:100, Abcam, Cambridge, MA, USA) overnight at 4 °C. The next day, cells were washed with PBS and incubated with bovine anti-rabbit IgG-FITC (1:200, Santa Cruz Biotechnology) secondary antibody for 2 h at room temperature. Cells were mounted on slides with mounting solution containing DAPI (Vector Laboratories, Burlingame, CA, USA), and cells were viewed under a confocal microscope system (LSM700, Olympus, Center Valley, PA, USA).

### Mouse model and experimental protocols

Animals were handled according to national and international guidelines in an animal facility accredited by the Association for Assessment and Accreditation of Laboratory Animal Care (AAALAC). The number of animals used was minimized, and all necessary precautions were fulfilled to mitigate pain and suffering. Protocols were approved by the Institutional Animal Care and Use Committee in Yonsei Biomedical Research Institute.

A total of 36 four- to five-week-old mice were randomized into three groups according to the injection materials: PBS, control adenovirus (dE1-k35), and decoy Wnt receptor-expressing adenovirus (dE1-k35/sLRP6E1E2). A single fraction of 15 Gy was delivered to the dorsal skin (dimension: 1.5 × 2 cm) of each mouse using an X-RAD320 irradiator, ensuring that > 90% of the prescribed dose would be limited to skin depth. Post-irradiation, PBS or virus was directly injected into each irradiated dorsal skin at 1, 3, 5, 7, 9, and 11 weeks. For each mouse, 5 × 10^10^ viral particles (VP)/ml was administrated. The mice were housed and observed for 16 weeks to allow toxic effects of radiation to develop. At 2, 4, 6, 8, 10, and 12 weeks after irradiation, digital photographs and laser Doppler flowmetry (Periflux system 5,000, Perimed AB, Jarfalla, Sweden) measurements were taken. At 4, 8, 12, and 16 weeks, mice were euthanized (n = 3 each week), and tissues were harvested for histological analysis.

### Clinical evaluation of mouse model

Gross changes of the skin after a single fraction irradiation of 15 Gy were observed, and complications related to irradiation such as erythema, dry desquamation, moist desquamation, ulceration, hair loss, and necrosis were recorded. Irradiated areas were marked on the dorsum of each mouse before radiation, and the marked areas were later assessed by digital photographs. The Scion (NIH-Scion Corp., Frederick, MD, USA) image program was used by two blinded observers to digitally measure each affected area. Using the program, the length of each image was converted to the actual length, and the surface area was calculated. Sequential changes in the irradiated area were analyzed.

To assess changes in blood flow within the irradiated area, the Periflux system 5,000 was used. Skin vascularity was assessed from the center of the irradiated area using laser Doppler flowmetry. Data were measured three times at 1-min intervals, and the mean values were obtained. Data are presented as perfusion units (PU).

### Histological changes in collagen fibers

To analyze the histology of collagen fibers, Masson’s trichrome staining was performed. The staining solution was prepared with a standard protocol.

### Quantification of type I collagen

Immunohistochemistry was performed to measure the amount of type I collagen. Slides were incubated overnight at 4 °C with rabbit anti-collagen I antibody (Abcam) and rabbit anti-fibronectin (Santa Cruz Biotechnology) diluted in blocking serum. After several stages of washing with PBS, the tissue sections were incubated with peroxidase-conjugated goat anti-rabbit polyclonal antibody (DAKO, Carpinteria, CA, USA) at a dilution of 1:200 in PBS for 30 min at 37 °C. After several washes in PBS, the signals on the tissues were revealed by incubating the tissues with diaminobenzidine in PBS for 10 min.

Semi-quantitative analysis of the synthesis of type I collagen was executed using MetaMorph image analysis software (Universal Image Corporation, Buckinghamshire, UK). Results are expressed as the average optical density (OD) of five different digital images.

### Statistical analysis

Each experiment was performed in triplicate. Results are expressed as mean ± standard error of the mean (SEM). Group results were compared by one-way analysis of variance, followed by post hoc Student’s *t*-test for unpaired observations or Bonferroni’s correction for multiple comparisons. *P* < 0.05 was considered significant.

## Supplementary information

Supplementary file1 (PDF 648 kb)

## Data Availability

The datasets generated during and analysed during the current study are available from the corresponding author on reasonable request.
