# Supplementary material for: Inhibition of Wnt signaling pathway suppresses radiation-induced dermal fibrosis
Source: Sci Rep. 2020 Aug 12;10:13594. doi: 10.1038/s41598-020-70243-3 (PMC7423922; doi:10.1038/s41598-020-70243-3)
Supplement: Supplementary file 1 — Supplementary file1 (PDF 648 kb) [file 41598_2020_70243_MOESM1_ESM.pdf]

Inhibition of Wnt Signaling Pathway Suppresses Radiation-Induced Dermal Fibrosis

Dong Won Lee, MD, PhD, Won Jai Lee, MD, PhD, Jaeho Cho, MD, PhD,

Chae-Ok Yun, PhD, Hyun Roh, PhD, Hsien Pin Chang, MD, Tai Suk Roh, MD, PhD,

Ju Hee Lee, MD, PhD, Dae Hyun Lew, MD, PhD

Supplementary Figure 1. Clinical results in a mouse model. **(a)** Gross changes in the skin. Tiny dry desquamations and hair loss were observed, but there were no ulcerations. No significant differences were noted between groups. **(b)** Planimetric analysis of irradiated area showed a gradual increase with time, but there was no significant difference between groups. ** $p<0.01$ vs sLRP6E1E2(-). **(c)** Cutaneous blood flow by laser Doppler flowmetry. Values were taken from the center of the irradiated areas. The values of the sLRP6E1E2-treated group are higher than those of other groups at 8 and 16 weeks post-radiation, but there was no significant difference between groups.

a

PBS

sLRP6E1E2 (-)

sLRP6E1E2 (+)

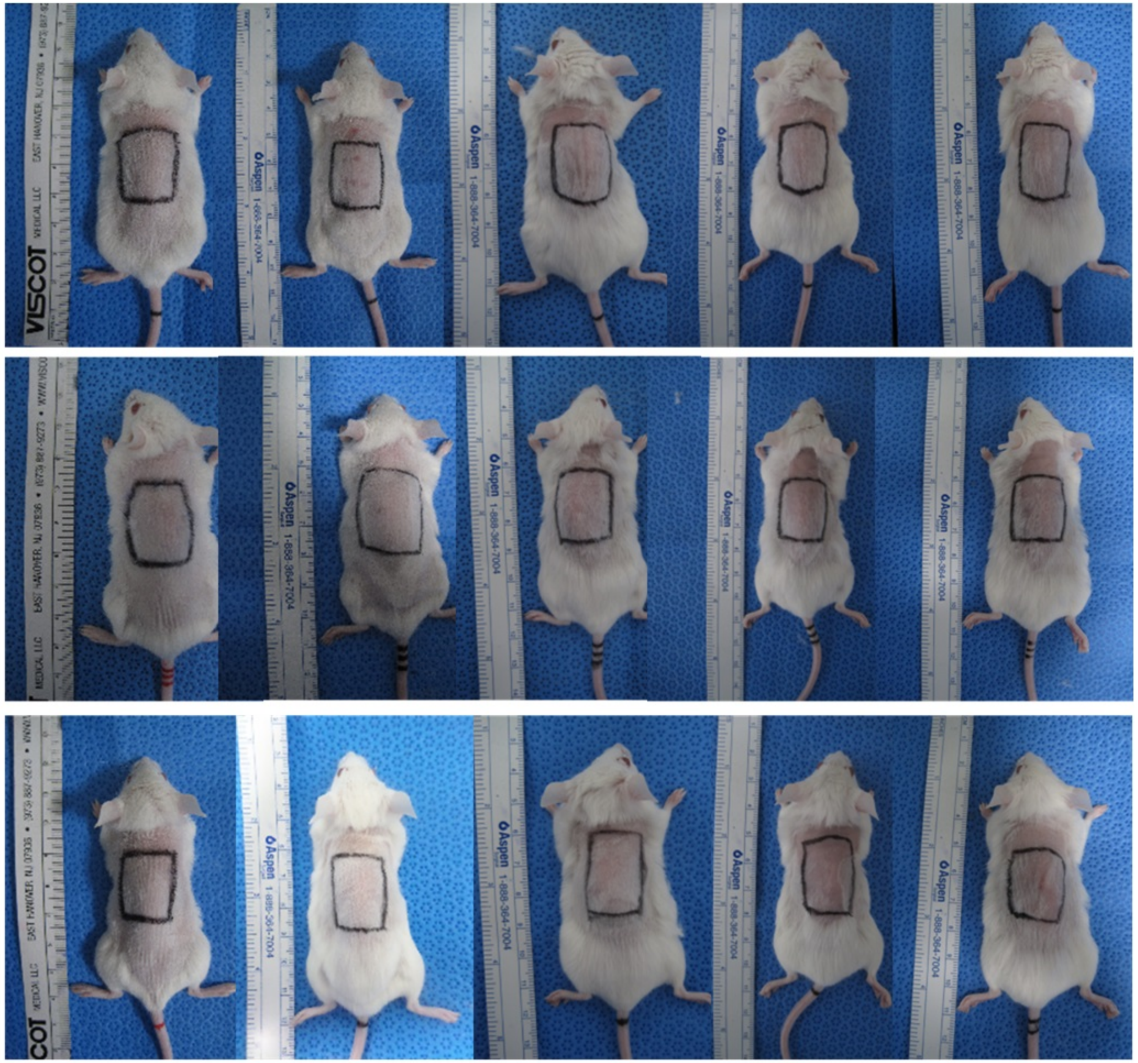

pre-radiation | 4 weeks | 8 weeks | 12 weeks | 16 weeks

b

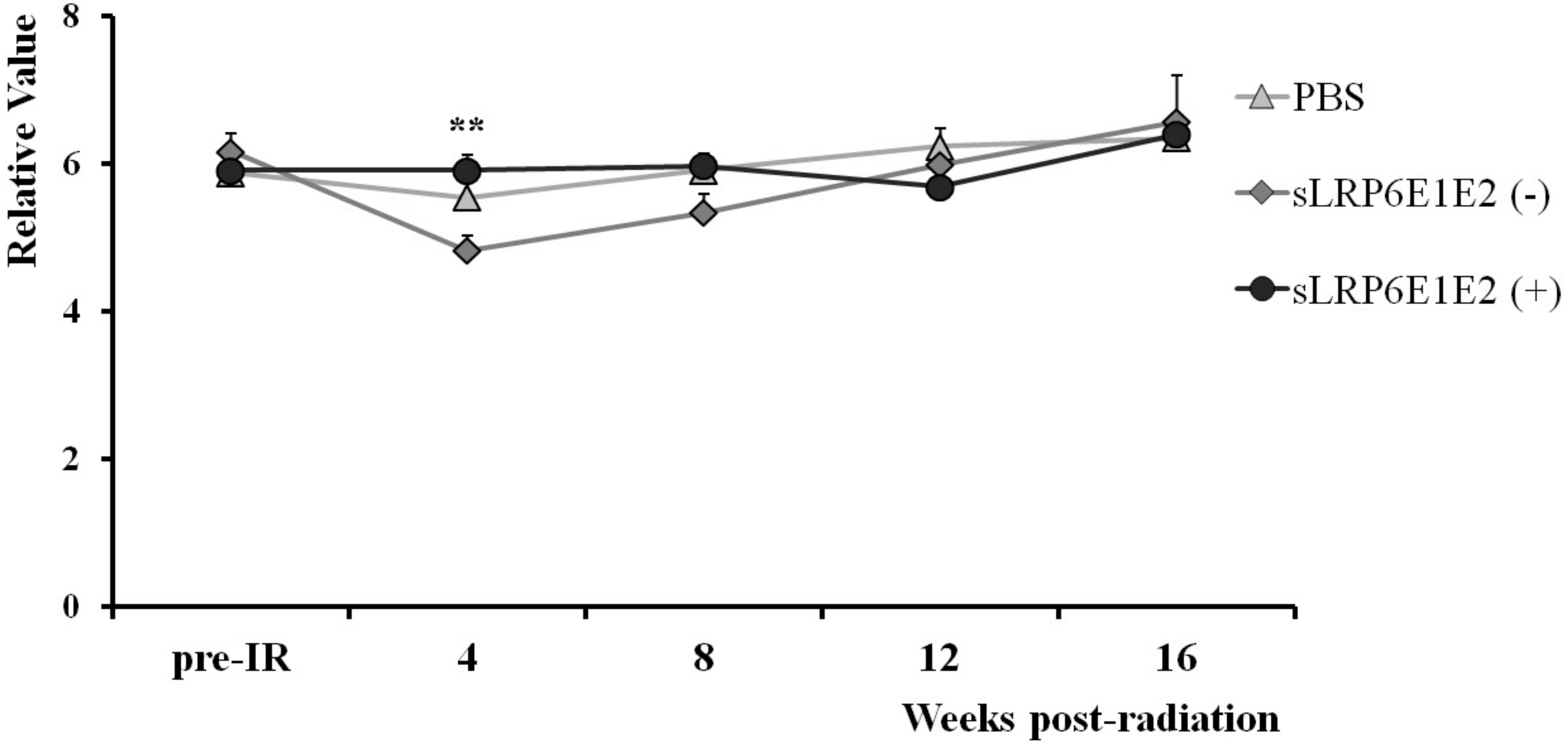
8
6
4
2
0
Relative Value
**
PBS
sLRP6E1E2 (-)
sLRP6E1E2 (+)
pre-IR
4
8
12
16
Weeks post-radiation

c

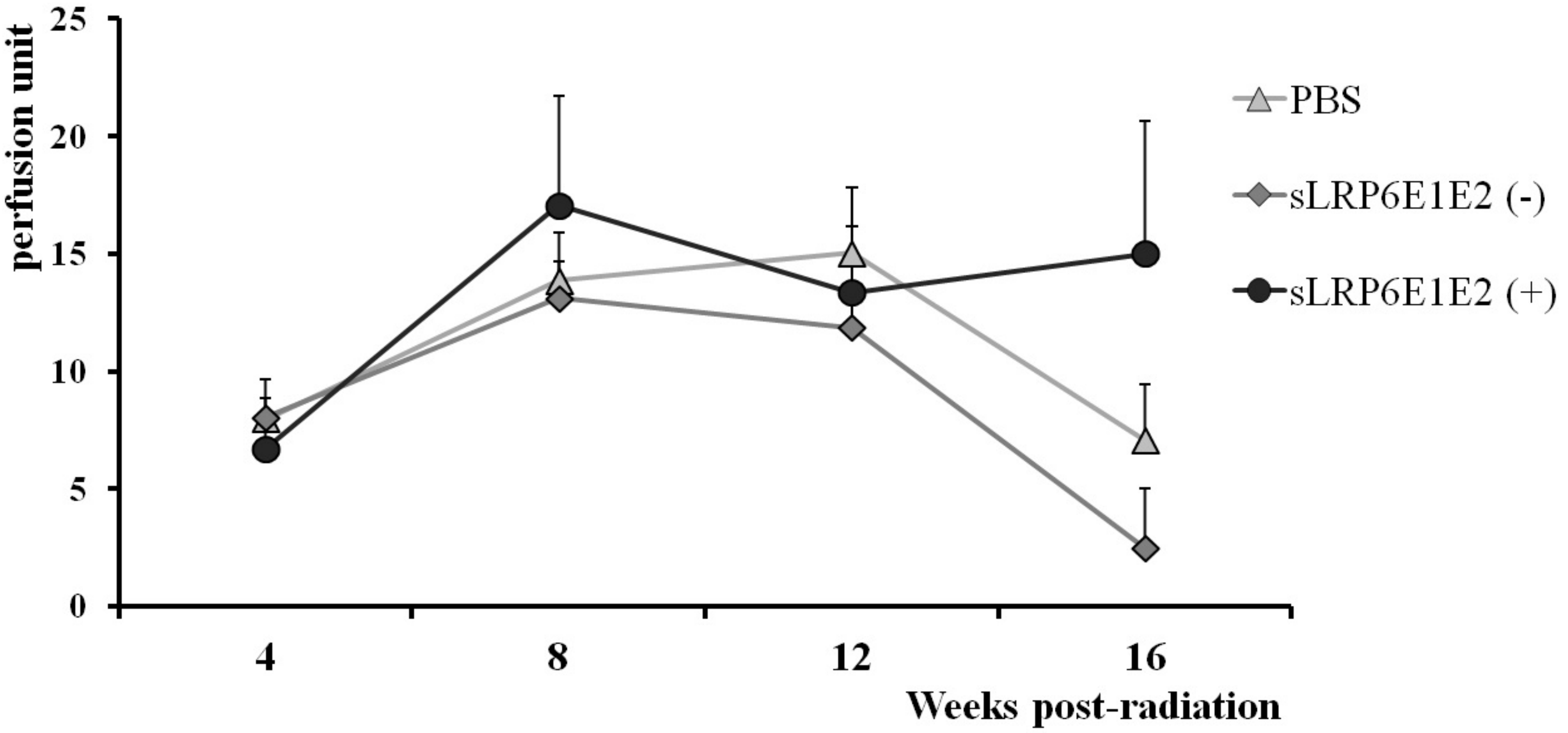

perfusion unit
25
20
15
10
5
0
4
8
12
16
Weeks post-radiation
PBS
sLRP6E1E2 (-)
sLRP6E1E2 (+)
